# Does Digital Screening for Atrial Fibrillation Reach its Target Group? Insights From the eBRAVE-AF Trial

**DOI:** 10.1016/j.jacadv.2025.102416

**Published:** 2025-12-11

**Authors:** Luisa Freyer, Peter Spielbichler, Lukas Tenbrink, Laura Elisa Villegas Sierra, Maria F. Vogl, Theofanis Korovesis, Mathias Klemm, Steffen Massberg, Axel Bauer, Konstantinos D. Rizas

**Affiliations:** a Medizinische Klinik und Poliklinik I, University Hospital Munich, Ludwig-Maximilians University, Munich, Germany; b Munich Heart Alliance, German Center for Cardiovascular Research (DZHK), Munich, Germany; c University Hospital for Internal Medicine III, Medical University of Innsbruck, Innsbruck, Austria

**Keywords:** atrial fibrillation, digital screening, photoplethysmography

**What is the clinical question being addressed?**

Does digital screening for atrial fibrillation reach its target group?

**What is the main finding?**

Digital screening for AF does not reach the high-risk group primarily in need of this technology.

Atrial fibrillation (AF) is the most common arrhythmia and is associated with increased risk of stroke and hospitalization.^1^ Several studies identified demographic parameters and cardiovascular conditions as risk factors for developing AF.^1^ Timely detection of AF can substantially reduce morbidity and mortality.^1^ Particularly the annual stroke risk, estimated by CHA_2_DS_2_-VA(Sc) score, can be significantly lowered through early initiation of oral anticoagulation (OAC). Current guidelines recommend considering the initiation of OAC in patients with estimated annual stroke risk of ≥1%, corresponding to a CHA_2_DS_2_-VA score of ≥1.^1^ Screening for AF has been proven effective not only in detecting unknown AF cases but also in identifying patients with known, untreated AF, which led to a significant net clinical benefit in previous studies.^2^ The adoption of smart-devices with pulse-measurement capabilities in everyday life has prompted the conduction of several digital AF-screening trials.^3^^,^^4^ However, these studies predominantly enrolled young participants without relevant comorbidities and a median CHA_2_DS_2_-VASc score of 1.^3^^,^^4^ High-risk participants with indication for OAC after detection of AF were underrepresented. Thus, enrollment of an elderly population that could benefit from digital AF screening remains a major challenge. eBRAVE-AF (eHealth-based Bavarian Alternative Detection of Atrial Fibrillation)^5^ was the first trial to randomly assess the efficacy of smartphone-based screening to detect treatment-relevant AF in direct comparison with usual care in an elevated-risk population. In this substudy, we investigated whether the digital AF-screening strategy effectively reached its intended population and found that the high-risk population was underrepresented.

In eBRAVE-AF, 67,488 policyholders of a German health insurance company (age 50-90 years, CHA_2_DS_2_-VA score ≥1, without known AF or OAC) were invited to participate. Of those invited, 5,551 individuals (8.2%) consented and were randomized to digital screening or usual care. For digital screening, participants used a photoplethysmography-based screening method to detect pulse irregularities. Abnormal findings were confirmed by Holter-electrocardiogram. The primary endpoint was newly diagnosed AF with initiation of OAC within 6 months. The type of OAC was at the discretion of the treating physician and both direct oral anticoagulants, as well as vitamin-K antagonists were allowed. After 6 months, 4,752 participants accepted the invitation to enter a second 6-month cross-over phase with reverse group assignment. In both phases, digital screening more than doubled the detection rate of treatment-relevant AF, compared to usual care.^5^

This substudy was approved by the local ethics committee of Ludwig Maximilian University of Munich Hospital (#23-0088) and is based exclusively on claims data (age, sex, arterial hypertension, diabetes mellitus, heart failure, previous stroke, and myocardial infarction), which correspond to the CHA_2_DS_2_-VA(Sc) score components and chronic kidney disease as an additional covariate. For regulatory reasons, these data were only available for 51,775 fully insured persons (77% of those invited). To account for baseline differences between participants and nonparticipants, we estimated propensity scores for study participation using generalized boosted regression modeling with participation as the response variable and the above clinical variables as covariates. The normalized inverse probability of treatment weights was calculated from propensity scores and was used to reconstruct a putative population with 51,775. The association between study group and the primary endpoint for both participants and the putative population was performed using Cox regression analysis, introducing the primary endpoint as dependent variable and the study group as time-dependent covariate. Comparison of HR between participants and the putative population was carried out by bootstrapping with replacement (N = 2,000 iterations). Event rates were extrapolated from Kaplan-Meier analysis. For the putative population, we fitted a Cox model to reconstruct the survival curve.

Nonparticipants were older (72 years; IQR: 65-78 years vs 65 years; IQR: 60-71 years), more frequently females (36 vs 31%), and suffered more frequently from hypertension (68% vs 25%; *P* < 0.001), diabetes mellitus (21% vs 5%; *P* < 0.001), stroke (2% vs 0%; *P* < 0.001), heart failure (12% vs 0%; *P* < 0.001), and myocardial infarction (25% vs 5%; *P* < 0.001) than participants. The annual event rate of major adverse cardiac or cerebrovascular events among participants was 2.3% (95% CI: 1.9%-2.7%), while the propensity-score matched extrapolated event rate in the entire population was significantly higher (3.4%; 95% CI: 3.2%-3.6%; *P* < 0.001). Digital screening in study participants significantly increased the annual rate of AF requiring OAC (2.7%; 95% CI: 2.1%-3.3%) compared to usual care (1.2%; 95% CI: 0.8%-1.6%; HR: 2.3; 95% CI: 1.5-3.6; *P* < 0.001). Prescribed OAC predominantly (99%) included factor Xa inhibitors. Propensity-score matched extrapolation of these results to all invited individuals showed that digital screening would be more than threefold more effective for detecting AF requiring OAC compared to usual care (3.4%; 95% CI: 3.2%-3.7% vs 1.0%; 95% CI: 0.9%-1.1%; HR: 3.6; 95% CI: 1.9-6.9; *P* < 0.001) (Figure 1). The difference between newly diagnosed AF requiring OAC between participants and nonparticipants was statistically significant (*P* = 0.008).

**Figure 1:**
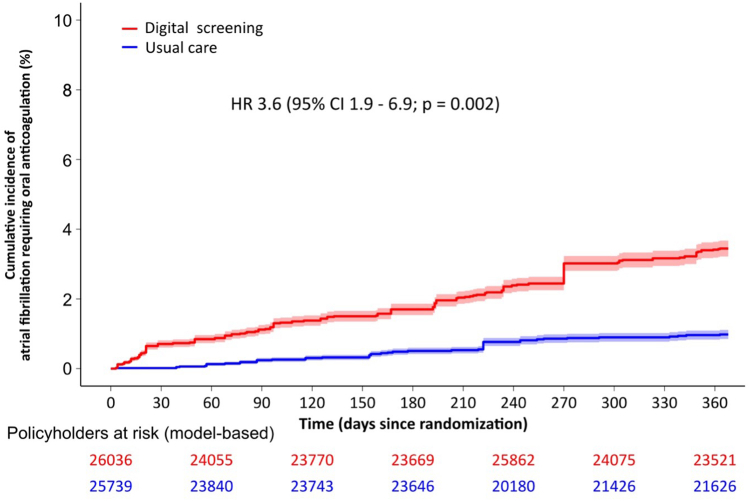
Detection of Atrial Fibrillation Requiring Oral Anticoagulation in a Model-Based Kaplan-Meier Analysis of 51,775 Policyholders Stratified by Screening Method

In summary, our analyses show that healthier persons with less comorbidities were more likely to participate in our digital trial. Older patients with a higher degree of comorbidities, who could substantially benefit from digital AF screening, were underrepresented. In a hypothesis-generating analysis using propensity-score matching, we showed that implementation of our screening protocol on a more vulnerable population would most likely lead to significantly higher detection rate of AF requiring OAC. Reaching the target population therefore remains an important unmet clinical need.

The main limitation of this study is that it was based on claims data, which were available in 77% of policyholders. Moreover, the estimated event rate of AF requiring OAC, as well as major adverse cardiac or cerebrovascular events for nonparticipants was based on mathematical models and might diverge from the real event rates. Second, the study was conducted in cooperation with a single German health insurance provider. Therefore, the study cohort might not be fully representative of the German or European population. Third, variations in smartphone use among older adults across regions may influence the generalizability of our results to other countries. Fourth, OAC indication in subclinical, digitally detected AF remains under debate, particularly in elderly patients, where risks-benefit considerations are complex and may be influenced by regional variations in drug availability and clinical practice. Our data do not permit conclusions about the impact of frailty on OAC-related adverse effects.

Despite these limitations, this substudy showed that digital AF screening did not reach the high-risk group primarily in need of this technology. Future AF screening studies should focus on accomplishing higher participation rates of more vulnerable individuals. Age-related barriers for usage of digital devices, such as visual impairments, can be partially overcome by simple and user-tailored device designs. Moreover, digital trials should focus on modern study protocols using dynamic, event-driven sample size calculations and randomization using adaptive stratification to ensure robust results across both healthier and more vulnerable populations.

## Funding support and author disclosures

The eBRAVE-AF study was primarily funded by resources of 10.13039/501100005722LMU Hospital, Munich, Germany. The study received partial funding by Pfizer Pharma GmbH. The PPG app was provided by Preventicus GmbH. The funders of the study have no role in the study design, data collection and analysis, data interpretation, and writing of the report. The authors have reported that they have no relationships relevant to the contents of this paper to disclose.
